# Development and validation of a LASSO logistic regression based nomogram for predicting live births in women with polycystic ovary syndrome: a retrospective cohort study

**DOI:** 10.3389/fendo.2025.1525823

**Published:** 2025-05-27

**Authors:** Yue Liu, Jingshu Gao, Hang Ge, Jiaxing Feng, Yu Wang, Xiaoke Wu

**Affiliations:** ^1^ Graduate School, Heilongjiang University of Chinese Medicine, Harbin, China; ^2^ The First Affiliated Hospital, Heilongjiang University of Chinese Medicine, Harbin, China; ^3^ The First Affiliated Hospital, Zhejiang Chinese Medical University, Hangzhou, China

**Keywords:** polycystic ovarian syndrome, live birth, lasso-logistic regression, nomogram, prediction model

## Abstract

**Objective:**

There is limited study on predictive models for live births in patients with polycystic ovarian syndrome (PCOS). The study aimed to develop and validate a nomogram for predicting live births in Chinese women with PCOS, as well as to identify the risk factors affecting live births in this population.

**Methods:**

Data for this study were obtained from a large clinical trial known as PCOSAct in mainland China. A total of 927 patients with PCOS were investigated using stratified random sampling. The mean-filling method was used to address missing data, and Lasso-Logistic regression was combined with machine learning models to identify the most significant predictors of live births. A nomogram was constructed based on a multivariate logistic regression model and was evaluated using receiver operating characteristic curves (ROC), calibration curves and clinical decision curves to assess model discrimination, calibration and clinical validity.

**Results:**

In the training set, estradiol, potassium ion, total cholesterol, alanine aminotransferase, and free androgen index were identified as key risk factors influencing live birth rates in PCOS patients. In this study, we demonstrated that elevated levels of estradiol and potassium ions, coupled with reduced levels of total cholesterol, alanine aminotransferase, and free androgen index (FAI), significantly improved insulin resistance (IR) in patients with polycystic ovary syndrome (PCOS). These biochemical alterations facilitated weight reduction and normalized endocrine function, thereby enhancing the live birth rate among PCOS patients. The nomogram developed from this multivariate model underwent validation by both internal (AUC:0.649; 95% CI [0.605~0.694]) and external (AUC:0.709; 95% CI [0.616~0.801]) assessments. The results have strong predictive accuracy, reliability, and generalizability for live birth.

**Conclusion:**

The nomogram developed in this study is a valid tool for assessing live births in patients with PCOS. This tool will assist clinicians in assessing the risks associated with live births in patients with PCOS and enable them to implement more effective preventive measures.

## 1 Introduction

Polycystic ovarian syndrome (PCOS) is a chronic condition characterized by abnormal hormone levels resulting from ovarian dysfunction. It is one of the most prevalent endocrine disorders among women of reproductive age (1), affecting 8%~13% of women worldwide (2). The most common clinical symptoms of PCOS include amenorrhea, insulin resistance (IR), hyperandrogenemia, and polycystic ovarian changes (3). These symptoms can lead to various complications, including infertility, obesity, cardiovascular issues, and disruptions in glucose and lipid metabolism (4). Furthermore, women with PCOS are at an increased risk for adverse pregnancy outcomes, such as preterm membrane rupture, miscarriage, pre-eclampsia, gestational diabetes, preterm labor, and macrosomia, compared to women without the condition (5, 6).

The primary objective of medical care for infertility in patients with PCOS is to achieve a live birth. However, the mechanisms influencing live birth rates in women with PCOS are not yet fully clear. Research (7) indicates that factors such as poor egg and embryo quality and endometrial hyperplasia significantly contribute to the lower live birth rates observed in these patients. Chronic inflammation of the ovaries and uterus may contribute to reproductive abnormalities (8). Furthermore, a retrospective study (9) examining women with PCOS who underwent *in vitro* fertilization/intracytoplasmic sperm injection (IVF/ICSI) identified several factors that act as independent predictors of live birth rates within this demographic. These factors include body mass index (BMI), anti-Müllerian hormone (AMH) levels, the initial dose of follicle-stimulating hormone (FSH), serum luteinizing hormone (LH) and progesterone levels on the day of human chorionic gonadotropin (HCG) administration, as well as endometrial thickness. Gunning et al. identified additional predictors associated with increased live birth rates among women diagnosed with PCOS. These predictors include being of white race, absence of smoking, lower BMI, reduced insulin and total testosterone levels, and elevated concentrations of sex hormone-binding globulin (SHBG) (10). Moreover, factors such as female age, duration of infertility, BMI, serum testosterone and progesterone levels on the day of HCG injection, as well as the number and quality of high-quality cleavage-stage embryos transferred, can also influence live birth outcomes in fresh embryo transfer cycles for patients with PCOS (11).

Currently, clomifene citrate (CC) is widely acknowledged as a first-line ovulatory agent in the clinical management of PCOS, aimed at enhancing pregnancy and live birth rates. This is accomplished through the stimulation of FSH secretion, which facilitates the maturation of ovarian follicles. A recent meta-analysis highlighted the substantial effectiveness of a combined therapeutic approach that incorporates CC, metformin (MET), and pioglitazone (PIO) in enhancing live birth rates among PCOS patients (12). However, it is important to acknowledge that participants in the study who received treatment with CC exhibited an increased risk of multiple pregnancies. Furthermore, the administration of PIO was associated with a higher incidence of miscarriages within this patient cohort. Conversely, treatment with inositol and MET has been shown to enhance oocyte and embryo quality, presenting a safer option with fewer side effects and potentially resulting in higher live birth rates (13, 14). In Xia et al, berberine has been proposed as a potential intervention for addressing IR and glycolipid metabolic disorders in patients with PCOS. Additionally, it may contribute to the reduction of inflammation in the ovaries and uterus, thereby potentially improving live birth rates (15).

While previous studies have developed prediction models for live births in PCOS patients, most have focused on analyzing risk factors for cumulative live birth rates in those undergoing assisted reproductive technology (9–11). Notably, no studies have yet created a nomogram prediction model for live birth probabilities in PCOS patients using the lasso-logistic regression method. Lasso-logistic regression incorporates a regularization term that shrinks some regression coefficients to zero, thereby directly performing feature selection. This allows our model to automatically exclude irrelevant or redundant variables, mitigating the risk of overfitting and enhancing both the interpretability and generalizability of the model. Therefore, this study aims to identify independent risk factors for predicting live births among 927 PCOS patients who received ovulation induction therapy, employing the lasso-logistic regression approach.

## 2 Patients and methods

### 2.1 Study design and population

This study is based on a randomized controlled clinical trial conducted in mainland China, known as the Acupuncture and Clomiphene in Polycystic Ovary Syndrome Trial (PCOSAct). The trial recruited 1,000 patients with PCOS who met the modified Rotterdam criteria (1) from 25 hospitals across 20 cities in China. The primary study design and findings have been successfully published (16, 17). The trial received approval from the Ethics Committee of the First Hospital Affiliated with Heilongjiang University of Traditional Chinese Medicine (Approval No. 2010HZYLL-010), and the clinical trial number is NCT01573858. In this study, we excluded 73 patients who discontinued treatment, and we retrospectively examined the clinical data of 927 patients with PCOS.

### 2.2 Clinical variables and live birth

This study investigated the impact of various factors, including anthropometric indices, sex hormone levels, glycolipid metabolism indices, liver and renal function parameters, and ion concentrations, on live birth outcomes in a cohort of 927 patients diagnosed with PCOS. Live birth is defined as the delivery of a viable infant. Anthropometric measurements and hormonal indicators for all participants were collected following an overnight fast, while biochemical analyses were conducted in a laboratory environment. The free androgen index (FAI) is calculated using the following formula: SHBG (nmol/L) ×100/total testosterone (nmol/L). The Homeostatic Model Assessment of IR (HOMA-IR) is calculated using the following formula: fasting glucose (mmol/L) × fasting insulin (μU/mL)/22.5.

### 2.3 Data analysis

A stratified sampling method was employed to ensure that the proportion of positive samples in the training and validation sets was comparable. The dataset was randomly divided into a training set (n=742) and a validation set (n=185) in an 8:2 ratio. Serum samples from the experimental subjects were uniformly collected and subsequently analyzed. During this procedure, a small number of serum samples failed to be successfully collected, which resulted in missing values for certain biochemical indicators in some patients. The overall proportion of missing data in this study was approximately 8.6%. Consequently, the mean padding method was utilized to address the missing variable values in this study. The mean imputation method is widely applicable and features a straightforward and efficient algorithm. It is particularly suitable for addressing the minor missing data in our dataset, thereby avoiding the reduction of sample information and potential biases. Variables that followed a normal distribution were statistically characterized using the mean ± standard deviation, whereas those did not conform to a normal distribution were described using the median and interquartile range. The characteristics of the two datasets was compared using the independent samples t-test and the Mann-Whitney U test.

In the training dataset, variables were screened utilizing Least Absolute Shrinkage and Selection Operator (LASSO) regression to mitigate the risk of model overfitting. The variables identified through LASSO regression were subsequently analyzed using both univariate and multivariate logistic regression techniques. A nomogram was developed to predict live births in patients with PCOS utilizing a multivariate model consisting of optimal predictors. The discriminative ability of the nomogram was assessed through receiver operating characteristic (ROC) curves, AUC value was the area covered by ROC curve. The generalizability of the nomogram was evaluated through 1,000 repetitions of calibration curves, while the Hosmer-Lemeshow (H-L) test was employed to assess the calibration curves, with a significance threshold set at *P < 0.05*. Finally, the clinical validity of the nomogram was appraised using a decision curve analysis (DCA).

**Figure 1:**
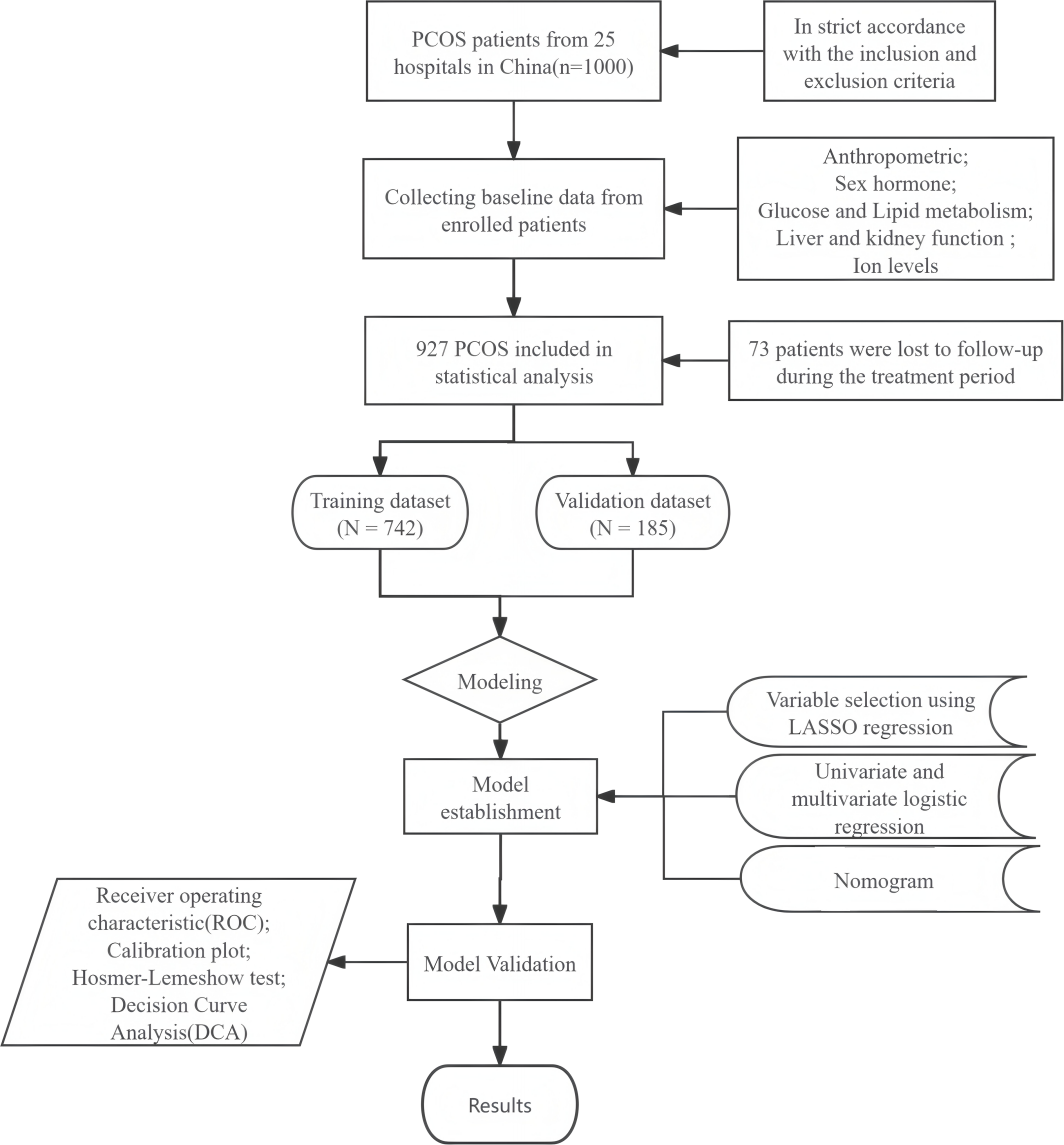
Study flowchart.

Statistical analyses were conducted utilizing SPSS software (version 27.0, USA), and R software (version 4.3.2, USA). A two-tailed analysis with a significance level of *P < 0.05* indicated that the observed differences were statistically significant. A comprehensive overview of the study's methodology can be seen in **Figure 1**.

## 3 Result

### 3.1 Participants’ characteristics

A total of 927 patients were enrolled in the study, comprising 742 individuals in the training dataset and 185 individuals in the validation dataset. **Table 1** presents the baseline characteristics of the cohort. A comparison of the baseline data revealed there minimal significant differences between the training and validation datasets regarding the general characteristics of the patients.

**Table 1:** Patient characteristics in the training and the validation dataset.

Variables	Total (N = 927)	Training dataset (N = 742)	Validation dataset (N = 185)	*P value*
Anthropometric				
Age (years)	28 (26, 30)	28 (26, 30)	28 (26, 30)	0.602
Height (cm)	161 (158, 165)	161 (158, 165)	160 (158, 165)	0.155
Weight (kg)	61 (54, 70.1)	62 (54.05, 71)	60 (53, 67.5)	0.063
BMI (kg/m^2^)	23.71 (21.02, 26.76)	23.74 (21.09, 27.09)	23.53 (20.79, 26.03)	0.144
Hip (cm)	98 (92, 103)	98 (93, 104)	96.5 (90, 102)	0.009
Waist (cm)	84 (77, 92.7)	85 (78, 93)	82 (75, 90)	0.031
WHR	0.86 (0.82, 0.91)	0.86 (0.82, 0.91)	0.85 (0.81, 0.91)	0.324
Systolic BP (mmHg)	110 (108, 120)	110 (108, 120)	110 (110, 120)	0.815
Diastolic BP (mmHg)	75 (70, 80)	75 (70, 80)	74 (70, 80)	0.533
Sex hormones				
Progesterone (nmol/L)	1.74 (1.24, 2.36)	1.74 (1.23, 2.34)	1.77 (1.33, 2.4)	0.116
LH (mIU/mL)	9.32 (6.22, 13.87)	9.32 (6.2, 13.95)	9.23 (6.24, 13.44)	0.708
FSH (mIU/mL)	6.02 (5.08, 6.98)	6.02 (5.09, 6.97)	6.02 (4.99, 7.03)	0.926
LH/FSH ratio	1.58 (1.08, 2.29)	1.58 (1.06, 2.28)	1.57 (1.09, 2.29)	0.784
Estradiol (pmol/L)	199 (159.15, 263.15)	199 (159.43, 264.1)	196.4 (158.8, 261.5)	0.875
SHBG (nmol/L)	33.7 (22.05, 53.5)	33.7 (22.02, 52.38)	36.7 (22.1, 57.4)	0.063
Total testosterone (nmol/L)	1.6 (1.21, 2.03)	1.6 (1.22, 2.04)	1.59 (1.2, 1.97)	0.526
Free testosterone (pmol/L)	2.21 (1.7, 2.77)	2.21 (1.69, 2.82)	2.21 (1.73, 2.59)	0.268
FAI	4.77 (2.73, 7.66)	4.77 (2.8, 7.76)	4.22 (2.5, 7.04)	0.04
Glucose and Lipid				
Total fasting insulin (pmol/L)	73.53 (48.11, 113.05)	73.53 (50.2, 115.01)	65.41 (41.03, 103.4)	0.007
Fasting glucose (mmol/L)	5.05 (4.58, 5.52)	5.05 (4.6, 5.52)	5.01 (4.46, 5.48)	0.221
HOMA-IR	2.32 (1.48, 3.66)	2.32 (1.54, 3.8)	2.01 (1.21, 3.34)	0.011
TC (mmol/L)	4.65 (4.01, 5.32)	4.65 (3.99, 5.33)	4.65 (4.11, 5.31)	0.805
TG (mmol/L)	1.31 (0.95, 1.93)	1.31 (0.97, 1.95)	1.24 (0.9, 1.76)	0.108
Lipoproteins A (g/L)	101.55 (69.6, 150.35)	101.55 (69.15, 147.5)	101.55 (75.1, 155.6)	0.531
HDL (mmol/L)	1.24 (1.02, 1.48)	1.24 (1.02, 1.46)	1.23 (1, 1.53)	0.895
LDL (mmol/L)	2.91 (2.41, 3.46)	2.91 (2.4, 3.47)	2.93 (2.43, 3.44)	0.738
Apolipoprotein A_1_ (g/L)	1.48 (1.3, 1.69)	1.48 (1.29, 1.68)	1.49 (1.33, 1.71)	0.717
Apolipoprotein B (g/L)	0.87 (0.7, 1.05)	0.87 (0.7, 1.06)	0.87 (0.7, 1.03)	0.778
Liver and renal function				
ALT (U/L)	7 (4, 10.5)	7 (4, 11)	7 (4, 9)	0.178
AST (U/L)	12 (9, 15)	12 (9, 15)	12 (9, 14)	0.262
Lactate dehydrogenase (U/L)	84 (52, 113)	84 (52, 112)	84 (54, 116)	0.449
Creatine Kinase (U/L)	51 (39, 63)	51 (39, 63.75)	51 (40, 62)	0.737
CK-MB (U/L)	2 (2, 2)	2 (2, 2)	2 (2, 2)	0.738
Urea (mmol/L)	4.2 (3.57, 4.96)	4.2 (3.55, 4.9)	4.22 (3.64, 5.14)	0.312
Creatinine (umol/L)	41.6 (35.35, 48.6)	41.6 (35.12, 48.5)	41.6 (36.7, 48.8)	0.405
Total bilirubin (umol/L)	5.8 (4.3, 7.55)	5.8 (4.3, 7.6)	5.8 (4.3, 7.3)	0.666
Direct bilirubin (umol/L)	2.1 (1.4, 2.9)	2.1 (1.4, 2.9)	2.1 (1.4, 2.8)	0.563
Indirect bilirubin (umol/L)	3.8 (2.9, 4.9)	3.8 (2.92, 4.9)	3.8 (2.9, 4.9)	0.671
Total bile acids (umol/L)	1.3 (0.7, 2.2)	1.3 (0.7, 2.2)	1.3 (0.8, 2.2)	0.857
Beta-2 microglobulin (mg/L)	1.25 (1.07, 1.46)	1.25 (1.07, 1.46)	1.25 (1.09, 1.44)	0.734
Homocysteine (umol/L)	7.53 (5.18, 10.05)	7.53 (5.22, 10.25)	7.49 (5.11, 9.3)	0.2
Cystatin C (mg/L)	0.46 (0.36, 0.54)	0.46 (0.36, 0.54)	0.46 (0.38, 0.54)	0.988
Ion levels				
Calcium (mmol/L)	1.97 (1.69, 2.2)	1.97 (1.68, 2.19)	1.97 (1.78, 2.21)	0.208
Phosphorus (mmol/L)	1.12 (0.99, 1.24)	1.12 (0.99, 1.24)	1.12 (0.99, 1.23)	0.74
Magnesium (mmol/L)	0.86 (0.77, 0.96)	0.86 (0.76, 0.96)	0.86 (0.79, 0.96)	0.293
Sodium (mmol/L)	136 (130, 143)	136 (129, 143)	136 (131, 143)	0.213
Potassium (mmol/L)	4.09 (3.82, 4.36)	4.09 (3.82, 4.35)	4.09 (3.82, 4.37)	0.844
Chloride (mmol/L)	94 (89, 99)	94 (88, 99)	94 (90, 98)	0.285

WHR, waist to hip ratio; TC, total cholesterol; TG, triglyceride; HDL, high-density lipoprotein; LDL, low-density lipoprotein; ALT, alanine aminotransferase; AST, aspartate aminotransferase; CK-MB, creatine kinase MB isoenzyme.

### 3.2 Identifying predictors

**Figure 2:**
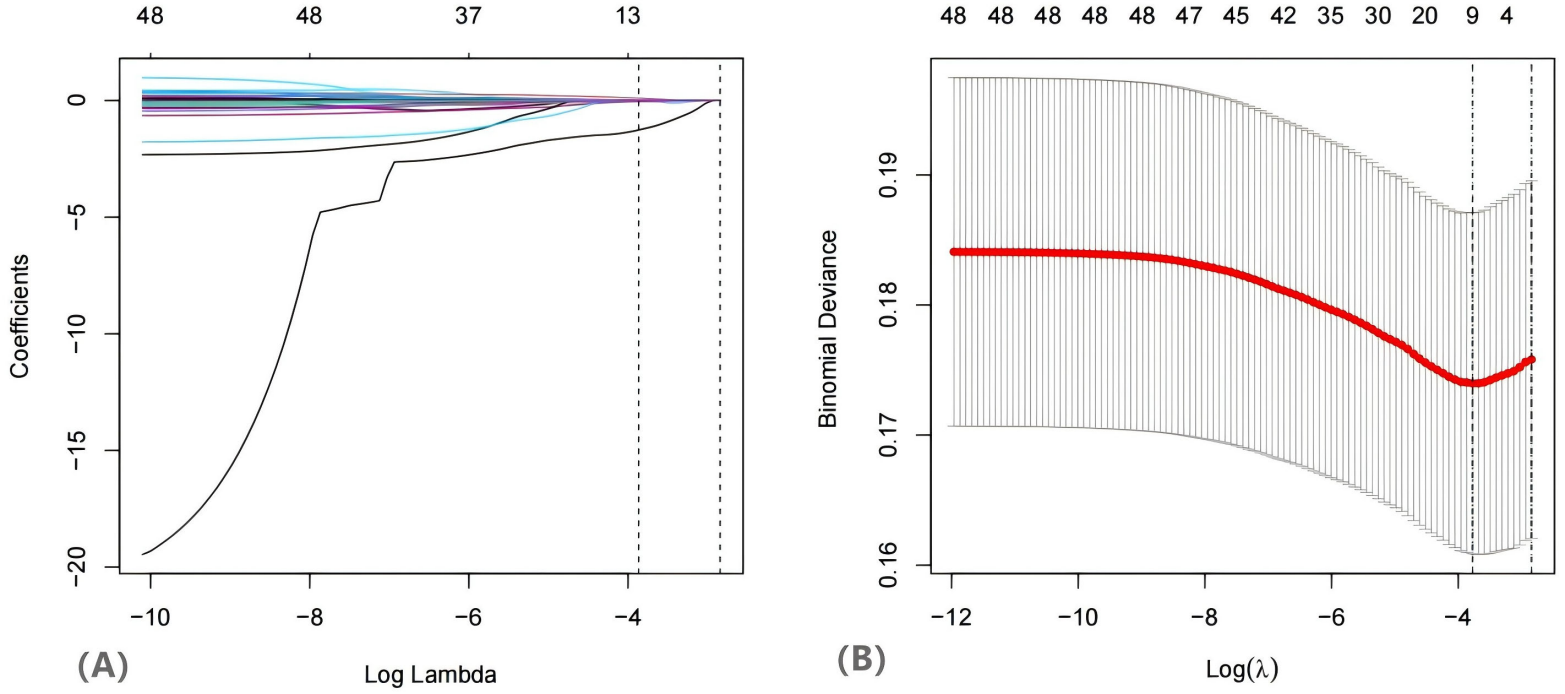
The variables filtering process of the Lasso regression. When birth was was used as endpoint. endpoint, Lasso regression suggested including 11 variables. Note: **(A)** Lasso coefficient profiles of the features. **(B)** Optimal parameter (lambda) selection in the Lasso model used tenfold cross validation via minimum criteria. The two dashed lines represent lambda.min and lambda.1se, lambda.min represents the λ value when the model error is the smallest, and lambda.1se represents the λ value within one standard error of the model error.

Among the 742 patients included in the training dataset, the occurrence of a live birth was designated as the dependent variable. The LASSO regression algorithm was employed to screen the variables (**Figure 2**). This method effectively minimized the impact of multicollinearity and demonstrated strong predictive capabilities along with high robustness. Independent features in within training set by were identified through coefficients in the LASSO regression. The selection of the parameter (lambda) for the LASSO model was determined through cross-validation based on established criteria.

In this study, the λ value corresponding to lambda.min was utilized as the screening criterion, as it yielded the lowest model prediction error, thereby ensuring that the model prediction constructed with the screened independent variables was the most accurate. Subsequently, the eleven variables identified through LASSO regression were incorporated into a univariate logistic regression analysis, where each variable underwent binary logistic regression. The statistically significant variables are presented in **Table 2**. Following the WHR, the variables estradiol, TG, TC, LDL, ALT, potassium ions (K^+^), FAI, and HOMA-IR were included in multivariate analysis (**Table 2**). The multivariate analysis indicated that ALT (OR =0.94, 95% CI [0.90, 0.97]), estradiol (OR = 1.01, 95% CI [1.01, 1.01]), TC (OR = 0.83, 95% CI [0.70, 0.99]), K^+^ (OR = 1.26, 95% CI [1.05, 1.51]), and FAI (OR = 0.93, 95% CI [0.88, 0.98]) were significant predictors of live birth outcomes.

**Table 2:** Univariate and multivariate logistic analysis of live birth in the training group.

Variables	Univariate logistic analysis					Multivariate logistic analysis				
	β	S.E	Z	*P*	OR (95%CI)	β	S.E	Z	*P*	OR (95%CI)
Age (years)	-0.05	0.03	-1.89	0.059	0.95 (0.90 ~ 1.00)					
WHR	-4.78	1.37	-3.48	**<0.001**	0.01 (0.00 ~ 0.12)					
FSH(mIU/mL)	0.09	0.06	1.62	0.105	1.09 (0.98 ~ 1.22)					
Estradiol (pmol/L)	0.01	0.00	2.08	**0.038**	1.01 (1.01 ~ 1.01)	0.01	0.00	2.00	**0.045**	1.01 (1.01 ~ 1.01)
TG (mmol/L)	-0.34	0.12	-2.93	**0.003**	0.71 (0.57 ~ 0.89)					
TC (mmol/L)	-0.24	0.09	-2.78	**0.005**	0.79 (0.66 ~ 0.93)	-0.19	0.09	-2.09	**0.036**	0.83 (0.70 ~ 0.99)
LDL(mmol/L)	-0.32	0.11	-2.93	**0.003**	0.73 (0.59 ~ 0.90)					
ALT(U/L)	-0.08	0.02	-3.98	**<0.001**	0.93 (0.89 ~ 0.96)	-0.06	0.02	-3.23	**0.001**	0.94 (0.90 ~ 0.97)
K^+^(mmol/L)	0.19	0.09	2.16	**0.030**	1.21 (1.02 ~ 1.43)	0.23	0.09	2.47	**0.013**	1.26 (1.05 ~ 1.51)
FAI	-0.09	0.03	-3.68	**<0.001**	0.91 (0.86 ~ 0.96)	-0.07	0.03	-2.62	**0.009**	0.93 (0.88 ~ 0.98)
HOMA-IR	-0.14	0.04	-3.11	**0.002**	0.87 (0.80 ~ 0.95)					

OR, Odds Ratio; CI, Confidence Interval; *P* < 0.05 was considered statistically significant. Bold represents P < 0.05.

### 3.3 Nomogram development and validation

**Figure 3:**
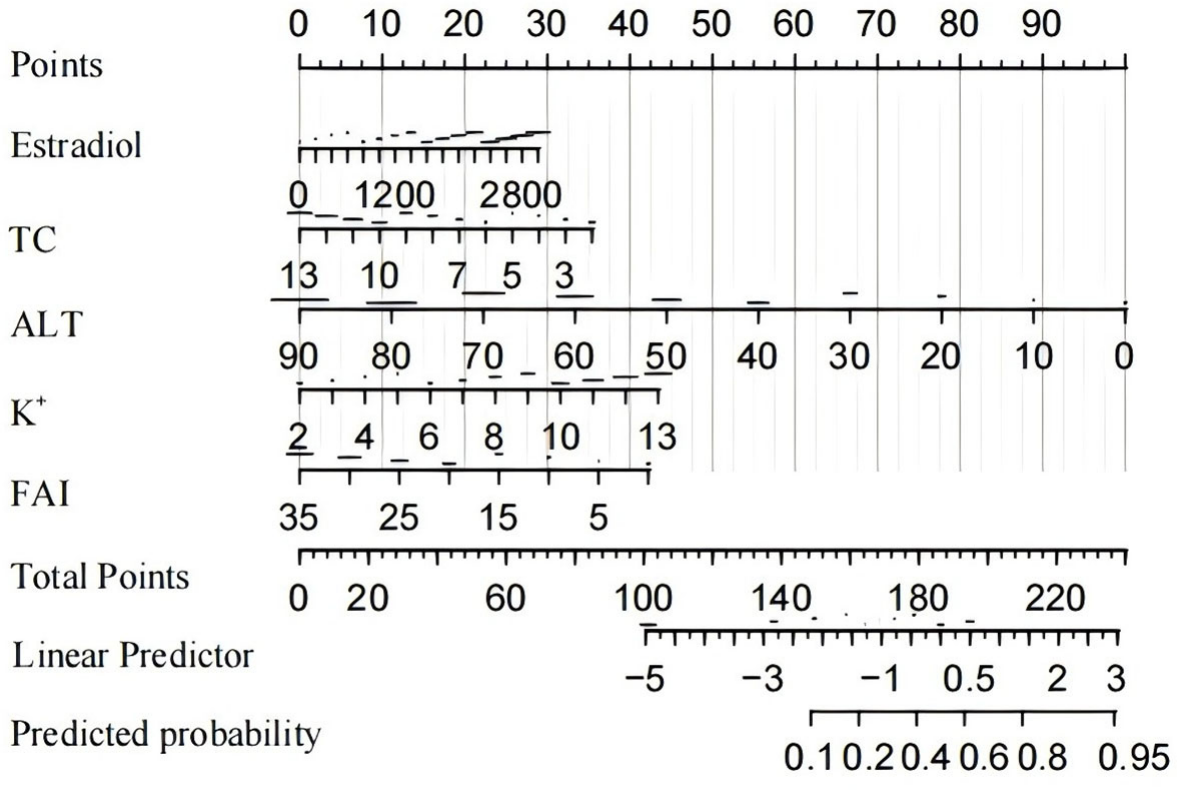
Nomogram for prediction of live birth and its predictive performance.

Based on the multivariate logistic regression analysis, five predictors were incorporated into the prediction model. Subsequently, we developed an individualized nomogram for predicting live birth outcomes in women with PCOS (**Figure 3**). The application of the nomogram is as follows: utilizing the nomogram, we can determine the points associated with each predictive indicator, and the cumulative points are recorded as the total score.

**Figure 4:**
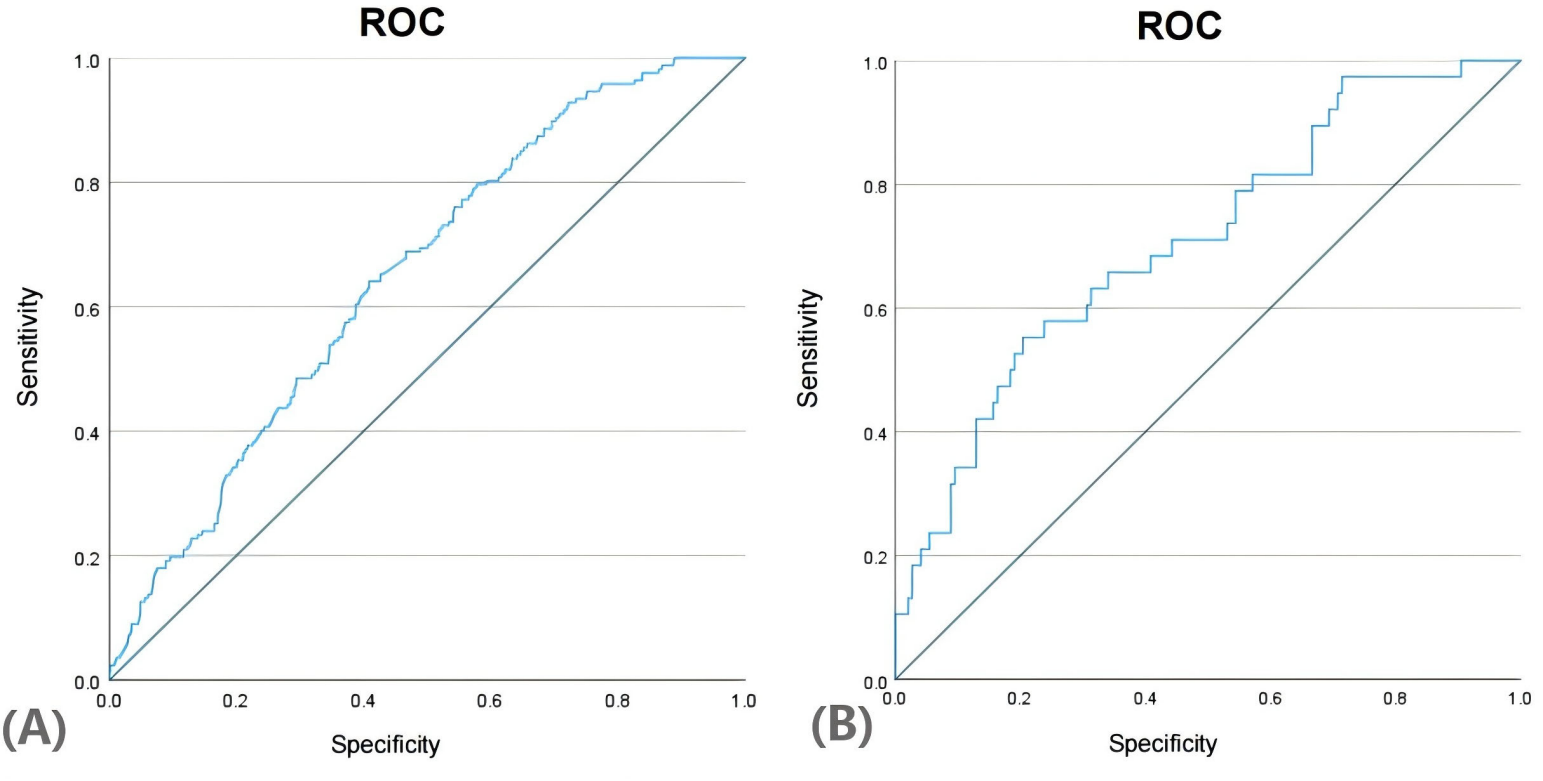
ROC curves in training group **(A)** and validation group **(B)**.

We generated the ROC curves for the predicted probabilities and calculated the AUC values for the predicted probabilities and calculated the AUC values for both the training and validation groups (**Figure 4**). The ROC curve was employed to derive the AUC values from models incorporating the five independent predictors included in the nomogram. The AUC values of the training group and validation group were 0.649 (95%CI:0.605~0.694) and 0.709 (95%CI:0.616~0.801), respectively. These results indicate that the nomogram prediction model may process useful discrimination ability.

**Figure 5:**
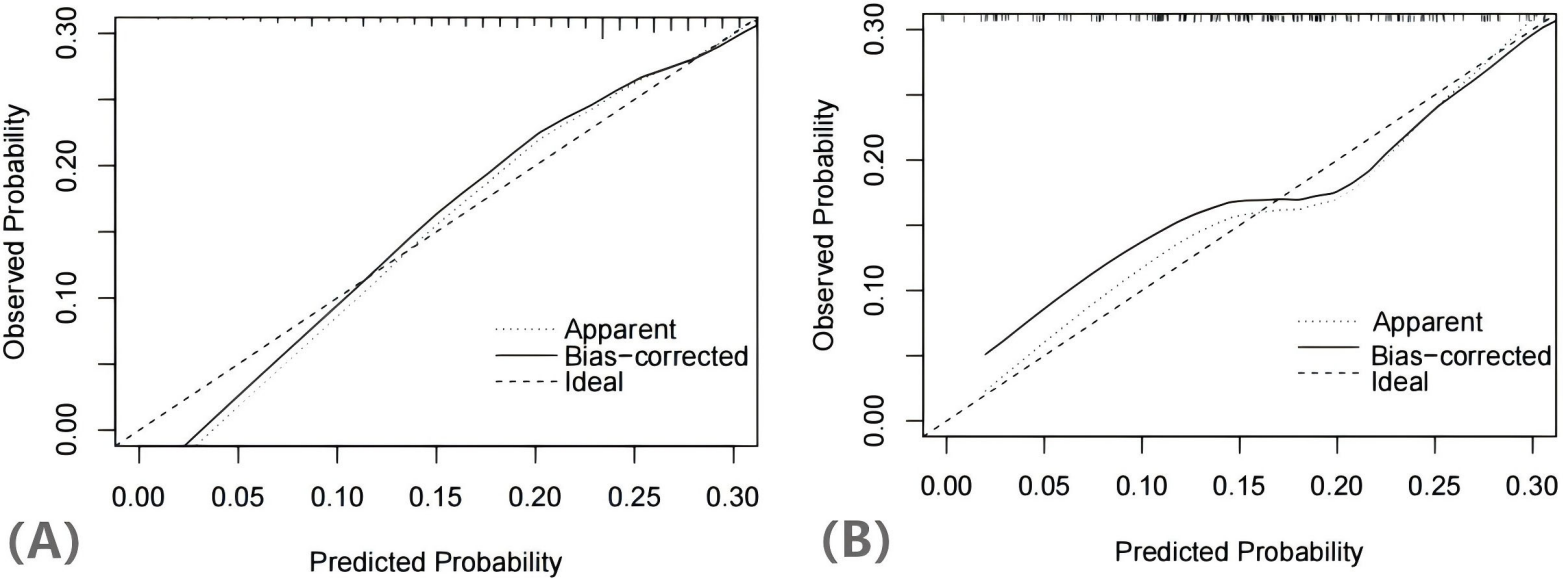
Calibration curves of the nomogram in training group **(A)** and validation group **(B)**.

**Figure 6:**
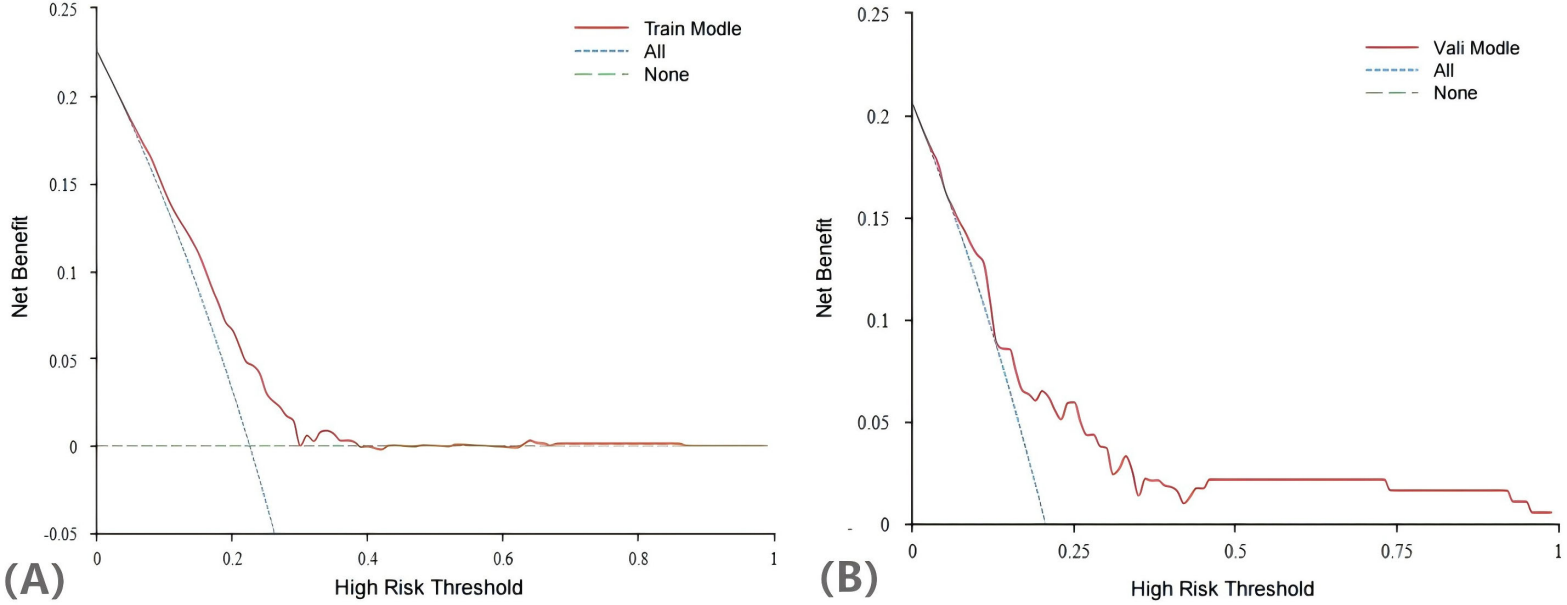
DCA curves of the nomogram in training group **(A)** and validation group **(B)**.

The H-L test yielded a χ^2^ statistic of 7.925 (*p*=0.441), indicating that the prediction model demonstrated good calibration (**Figure 5**). Additionally, the decision curves for DCA probability indicated that the net benefit for the training groups was maximized when the probability ranged between 15% and 35% (**Figure 6**).

## 4 Discussion

PCOS not only significantly impacts women’s physical health but also imposes considerable economic and psychological burdens on those affected. The live birth rate, a critical pregnancy outcome, is of substantial clinical importance. This study categorized PCOS patients based on their live birth status and examined how anthropometric measurements and relevant biochemical indicators influence live birth rates in this population. In our analysis of a training set comprising 742 patients, we found that elevated estradiol and potassium levels, along with reduced TC, ALT levels and FAI, significantly influenced live birth outcomes. Notably, we were the first to apply lasso-logistic regression to develop a nomogram prediction model for live births in PCOS patients, enhancing the model’s generalizability and preventing overfitting. To address missing data, we employed mean-padding method and included a larger number of continuous predictors to mitigate the negative effects of direct dichotomisation on predictive model accuracy. Additionally, we provided a visual representation of the risk factors affecting live birth outcomes in this cohort. Finally, we assessed the clinical utility of the model through decision curve analysis, aiming to inform healthcare professionals and health systems about the risk factors impacting live birth rates in patients with PCOS.

According to a multifactorial logistic regression analysis, estradiol and FAI were identified as independent risk factors for live birth in patients with PCOS. During IVF treatment (18), estradiol has been recognized as a predictor of pregnancy and live births. Moderate levels of estradiol can promote the maturation of female oocytes, resulting in the formation of healthy eggs, as well as to promote the thickening and preparation of the endometrium, thereby creating favourable conditions for the implantation of fertilised eggs in individuals with PCOS. In patients with PCOS, the ovaries have been observed to synthesize excessive amounts of androgens, which leads to the recruitment of multiple small follicles. However, these small follicles often do not respond adequately to normal concentrations of FSH, thereby hindering the formation of healthy eggs and preventing normal ovulation. Additionally, an inflammatory environment within the endometrium, exacerbated by elevated androgen levels and IR in PCOS patients, may impede the proliferation and differentiation of endometrial cells, thus obstructing the implantation of embryos (19). It has been noted that elevated estradiol levels in PCOS patients could contribute to an increased live birth rate by enhancing the endocrine environment and mitigating the adverse effects of androgens. The FAI is utilized to measure the levels of active androgens in the body. FAI as a sensitive predictor of reproductive outcomes in PCOS patients, showing significant associations with clinical pregnancy, ovulation per cycle, positive HCG tests, and live birth rates (20). In a retrospective cohort research, Zhao found that PCOS patients with elevated FAI exhibited levels lower rates of clinical pregnancies and live births (21).Our findings further imply that increased FAI levels negatively impact the live birth rate in PCOS patients. This phenomenon might be attributed to the heightened levels of IR in this population, which could be linked to FAI. Further, excessive testosterone levels may adversely affect oocytes quality, endometrial receptivity, and embryo implantation, thereby contributing to the reduced live birth rates in PCOS patients.

The live birth rate among patients with PCOS was found to be inversely correlated with both TC and ALT levels. In a study involving 178 PCOS patients undergoing IVF/ICSI, Gao et al. reported a significant negative connection between serum TC levels and live birth rates in PCOS patients (22). Additionally, research conducted by Jiang et al. identified serum TC levels as an independent risk factor for live birth rates in women with PCOS who underwent frozen-thawed embryo transfers, corroborating the findings of the present investigation (23). TC is a crucial component of cell membranes and is involved in hormone synthesis, thus, the dysregulated lipid metabolism associated with elevated TC levels may adversely affect ovarian function and follicular development, ultimately reducing the live birth rate in PCOS patients. Furthermore, previous studies have indicated that in patients undergoing IVF, the likelihood of live birth decreased with each standard deviation increase in ALT levels, with the live birth rate in the high-ALT group being significantly lower than that in the low-ALT group (24). The result showed that elevated ALT levels are indicative of abnormal fat accumulation in the liver and a diminished capacity for lipid metabolism, which can lead to abdominal obesity and fatty liver disease. This obesity may exacerbate the inflammatory response associated with IR, thereby negatively impacting ovarian function and the endometrial environment in PCOS patients, which in turn diminishes the success of embryo implantation and live birth rates.

The present study indicates a positive correlation between K^+^ levels and live birth rates in PCOS patients. Potassium ions are the predominant cations in the body and maintain the resting membrane potential and intracellular osmotic pressure of cells. Cem et al. observed that low serum potassium levels adversely affect insulin secretion in pancreatic endocrine β-cells, thereby triggering IR and exacerbating the symptoms of PCOS (25). Furthermore, Chu et al. demonstrated that higher intakes of magnesium and potassium may be associated with reduced body fat in individuals with impaired glucose tolerance, while insufficient dietary mineral intake could lead to obesity and metabolic diseases (26). Therefore, elevated serum potassium ion levels in PCOS patients may enhance insulin sensitivity and improve live birth rates.

In addition, we found through univariate regression that the WHR, TG, LDL, and HOMA-IR serve as predictive factors for live birth rates in patients with PCOS. Previous research had shown that elevated levels of TG, LDL, and HOMA-IR are independently related to a reduced live birth rate in individuals with PCOS (27). Furthermore, WHR has been identified as a more robust predictor of live births, pregnancies, and conceptions among this population (28). Meanwhile, the combination of WHR with HDL or TG may be utilized as a reliable diagnostic biomarker for metabolic syndrome (MS) in Chinese patients with PCOS (29), and there was an association between MS and a reduced live birth rate in PCOS patients (30). According to a clinical trial (31), women with PCOS who also presented with MS experienced prolonged infertility, as well as a decline in ovarian response, oocyte competence, and IVF live birth rates. Moreover, in women with PCOS undergoing ovulation induction, the presence of MS was associated with decreased live birth rates (32). This phenomenon is likely attributable to the negative effects of obesity and IR, which are consequences of MS and adversely impact ovulation and pregnancy outcomes in women with PCOS. Furthermore, a retrospective study found that (33) patients with PCOS and MS typically exhibited lower-quality embryos and an increased concentration of Fetuin-A in the follicular fluid, which negatively impacted embryo and, consequently, live birth rates. Ultimately, live birth rates in PCOS patients were adversely affected by changes in the maternal metabolic environment, which were directly linked to diminished oocyte quality, impaired endometrial function, and related unfavorable reproductive outcomes (34). In our study, while WHR, TG, LDL, and HOMA-IR were not incorporated into the multivariate regression analysis, they nonetheless exhibited a significant negative association with live births in patients with PCOS in clinical contexts. Consequently, it is imperative to undertake future clinical trials with larger sample sizes to further investigate the complex mechanisms that connect these metabolic markers to the outcomes of live birth in PCOS patients.

In contemporary clinical practice, oral contraceptive pills have emerged as a pivotal therapeutic modality for patients with polycystic ovary syndrome (PCOS). By elevating estrogen levels, oral contraceptives effectively suppress androgen levels, thereby promoting follicular development and maturation. This, in turn, significantly enhances the live birth rate among PCOS patients (35). Additionally, the long-term use of diabetes medications, such as metformin, has demonstrated substantial therapeutic benefits. Metformin not only effectively lowers blood glucose levels but also reduces body weight and improves ovulation, both of which contribute to increased live birth rates (36). Moreover, statins are frequently employed in clinical settings to reduce cholesterol levels and ameliorate glucose and lipid metabolic disorders in PCOS patients, further augmenting the live birth rate (37).

Beyond pharmacological interventions, lifestyle and nutritional modifications constitute the first-line treatment strategy for PCOS patients (38). Supplementation with potassium and magnesium ions in the diet has been shown to enhance insulin sensitivity (26). Furthermore, increasing exercise frequency and optimizing daily routines are equally critical. These measures collectively improve glucose-lipid metabolism and restore normal endocrine function, thereby creating favorable conditions for enhancing the live birth rate.

Compared with other models for predicting live births in PCOS patients, our Lasso-Logistic regression-based approach effectively mitigated issues related to overfitting of variables, facilitated the simultaneous analysis of all independent variables, demonstrated computational efficiency, and exhibited high modelling stability. In addition, the subjects of this study were derived from extensive clinical trials, which screened PCOS patients according to strict inclusion criteria, resulting in a population with considerable homogeneity. Consequently, the clinical credibility and practical applicability of the prediction model was high. In this study, we utilized DCA to evaluate the clinical utility of our predictive model for live-birth outcomes in PCOS patients. DCA is a valuable tool that helps assess the potential impact of a model in a clinical setting by comparing the net benefit of different risk prediction strategies across a range of probability thresholds. The DCA curve of the training set indicates that as the high - risk threshold rises, the model’s net benefit shows a downward trend. This means the model can yield positive net benefits across various high - risk thresholds. Moreover, within a broad range of thresholds, the model’s net benefit surpasses that of the “treat All” and “treat None” strategies. This implies that clinicians, when using this model for decision - making, can effectively identify patients most likely to benefit from interventions, thereby enhancing patient outcomes. The validation set results further substantiate the model’s reliability, showing that it can effectively predict live birth outcomes in PCOS patients and is applicable in real world clinical settings.

However, there were still some limitations in this study. Firstly, the sample size utilized in our prediction model was relatively limited, and due to the constraints imposed by the actual sample size,we did not employ external validation methods during model construction, which may introduce a degree of bias. Secondly, during the model development process, we used the mean padding method to address missing data, which could potentially contribute to the risk of bias in the results. Our study, a secondary analysis of a large - scale RCT, is affected by factors like sample size and statistical methods, so the model’s AUC value is not ideal. However, the model shows good calibration and clinical decision - making ability. It can serve as a preliminary screening tool for live births in PCOS patients, but it also has certain limitations. Therefore, future research should aim to further validate this prediction model with data from larger clinical trials, it is essential to continue collecting evidence from other centers and confirm the reliability of the model through external validation. In our subsequent studies, we will investigate the impact of lifestyle and psychological factors on the live birth rate among women with PCOS. This exploration will go hand - in - hand with our ongoing quest for a more diverse set of variables that could potentially hold stronger predictive power for live births related to PCOS. By doing so, we aim to expand the diagnostic value window and enhance the diagnostic capabilities of our model. Additionally, we should explore more sophisticated machine learning techniques and data imputation methods to reduce the risk of bias in the model, such as Random Forest, Support Vector Machine, and Decision Tree.

Our study combines traditional regression analysis methods with machine learning models to explore the factors affecting the live birth rate of PCOS more accurately. In addition, we developed a visual and personalized nomogram for predicting live birth outcomes, which demonstrated enhanced discrimination and calibration. It helps doctors and clinicians to provide a scientific basis for developing effective preventive and intervention measures to improve the live birth rate of PCOS patients.

## Data Availability

The raw data supporting the conclusions of this article will be made available by the authors, without undue reservation.
